# A longitudinal evaluation of oxidative stress - mitochondrial dysfunction - ferroptosis genes in anthracycline-induced cardiotoxicity

**DOI:** 10.1186/s12872-024-03967-z

**Published:** 2024-07-10

**Authors:** Ren Qianqian, Zhu Peng, Zhang Licai, Zhang Ruizhi, Ye Tianhe, Xia xiangwen, Zheng Chuansheng, Yang Fan

**Affiliations:** 1 Department of Radiology, Union Hospital, Tongji Medical College, Huazhong University of Science and Technology, Wuhan, 430022 China; 2 Hubei Province Key Laboratory of Molecular Imaging, Wuhan, 430022 China; 3 Department of Hepatobiliary Surgery, Wuhan No. 1 Hospital, Wuhan, China

**Keywords:** Cardiotoxicity, Anthracyclines, Oxidative stress, Mitochondrial dysfunction, Ferroptosis, Immune system, Angiogenesis, Drug side-effect

## Abstract

**Background:**

Antineoplastic medications, including doxorubicin, idarubicin, and epirubicin, have been found to adversely affect the heart due to oxidative stress - mitochondrial dysfunction - ferroptosis (ORMFs), which act as contributing attributes to anthracycline-induced cardiotoxicity. To better understand this phenomenon, the time-resolved measurements of ORMFS genes were analyzed in this study.

**Methods:**

The effect of three anthracycline drugs on ORMFs genes was studied using a human 3D cardiac microtissue cell model. Transcriptome data was collected over 14 days at two doses (therapeutic and toxic). WGCNA identified key module-related genes, and functional enrichment analysis investigated the biological processes quantified by ssGSEA, such as immune cell infiltration and angiogenesis. Biopsies were collected from heart failure patients and control subjects. GSE59672 and GSE2965 were collected for validation. Molecular docking was used to identify anthracyclines’s interaction with key genes.

**Results:**

The ORMFs genes were screened in vivo or in vitro. Using WGCNA, six co-expressed gene modules were grouped, with MEblue emerging as the most significant module. Eight key genes intersecting the blue module with the dynamic response genes were obtained: CD36, CDH5, CHI3L1, HBA2, HSD11B1, OGN, RPL8, and VWF. Compared with control samples, all key genes except RPL8 were down-regulated in vitro ANT treatment settings, and their expression levels varied over time. According to functional analyses, the key module-related genes were engaged in angiogenesis and the immune system pathways. In all ANT-treated settings, ssGSEA demonstrated a significant down-regulation of angiogenesis score and immune cell activity, including Activated CD4 T cell, Immature B cell, Memory B cell, Natural killer cell, Type 1 T helper cell, and Type 2 T helper cell. Molecular docking revealed that RPL8 and CHI3L1 show significant binding affinity for anthracyclines.

**Conclusion:**

This study focuses on the dynamic characteristics of ORMFs genes in both human cardiac microtissues and cardiac biopsies from ANT-treated patients. It has been highlighted that ORMFs genes may contribute to immune infiltration and angiogenesis in cases of anthracycline-induced cardiotoxicity. A thorough understanding of these genes could potentially lead to improved diagnosis and treatment of the disease.

**Supplementary Information:**

The online version contains supplementary material available at 10.1186/s12872-024-03967-z.

## Introduction

Anthracyclines (ANTs) are widely utilized in the clinical treatment of human malignancies and are generated from *Streptomyces peucetius var. caesius* [1]. Doxorubicin (Dox), epirubicin (Epi), and idarubicin (Ida) are typical ANTs. However, anthracycline-induced cardiotoxicity (AIC) develops in a dose-dependent cumulative pattern and results a range of cardiovascular outcomes, rapidly attracting attention [2, 3]. In a prospective follow-up of 2625 adult patients receiving anthracyclines, Cardinal et al. reported a 9% incidence of cardiotoxicity. In 98% of cases, AIC was identified during the first year following the end of treatment [4].

Numerous types of research have clarified the fundamental mechanisms of AIC, with oxidative stress triggered by an imbalance between the generation and neutralization of reactive oxygen species (ROS) being the most frequently reported molecular mechanism [5, 6]. Higher ROS levels generated by ANTs’ direct interaction with the mitochondrial electron transport chain [7] exceed the antioxidant capacity and damage cardiac tissue susceptible to free radical damage [8]. However, the combination of ANTs with various antioxidants, such as manganese superoxide dismutase (Mn-SOD) and glutathione peroxidase 1 (GPx1), has inconsistent cardioprotective effects [9–11], and therefore AIC has a complex and multifactorial pathophysiology.

The metabolic headquarters of cells and tissues, mitochondria, are significant generators and targets of oxidative stress [12, 13]. Recent research has suggested that AIC could have a probable etiology related to mitochondrial malfunction. ANTs-induced mitochondrial dysfunction leads to cell death through different mechanisms, including altering the mitochondrial respiratory chain, energy production, and mitochondrial dynamics and inducing mitochondrial oxidative/ nitrosative stress [14]. ANTs accumulation in mitochondria may mechanistically drive the OXPHOS complexes uncoupling [15, 16]. This occurs due to the mechanism by which ANTs interact with cardiolipin, a unique phospholipid of the inner mitochondrial membrane, thereby reducing ATP synthesis and boosting ROS generation.

The changes that occur in mitochondria due to oxidative stress are a major factor in developing ferroptosis. Accordingly, mounting evidence shows that mitochondria-dependent ferroptosis, triggered by iron overload and excessive lipid peroxidation, is crucial in developing AIC [17]. Ferroptosis highlighted by iron-dependent lipid peroxidation culminating in membrane rupture, is a newly defined nonapoptotic programmed cell death type [18–20]. ANTs accumulate in mitochondria by embedding into mtDNA in cardiomyocytes, disrupting iron utilization, and leading to iron overload; therefore, ANTs and iron accumulation synergistically trigger ferroptosis [20, 21]. Iron overload is typically linked to oxidative stress, which is defined by elevated levels of ROS and reduced levels of antioxidant enzymes [22], such as tetrahydrobiopterin (BH4), coenzyme Q10 and glutathione (GSH). GSH functions as the primary antioxidant in mammalian cells, essential for intracellular and extracellular equilibrium and ferroptosis [23]. In brief, reduced GSH levels lead to the synthesis of phospholipid hydroperoxides (PLOOH), which contribute to ROS accumulation and ferroptosis. Targeting ferroptosis, such as Fer-1, holds promise as a novel therapeutic strategy for cardioprotection [17, 24].

The cause-and-effect connection between oxidative stress and mitochondrial dysfunction in ferroptosis remains controversial, even though ferroptosis strongly contributes to AIC. Mitochondrial dysfunction contributes to stress responses as the cellular site of ROS production, such as the integrated stress response and the nuclear factor erythroid 2-related factor 2 (NRF2)-mediated oxidative stress response, which confer cellular resistance to oxidative stress and ferroptosis [20]. Previous research has typically concentrated on an individual gene or a prominent pathway involved in the development of AIC. However, the cumulative effect of a gene sequence and its interplay affected AIC. Moreover, the temporal relationship between these events has not been characterized. Therefore, there is an urgent need to create an integrated and efficient tool that precisely captures the characteristics of oxidative stress - mitochondrial dysfunction - ferroptosis (ORMFs). For the current research, the dynamic alteration of the oxidative stress - mitochondrial dysfunction - ferroptosis genes were unveiled in an in vitro cardiac microtissue exposed to 3 ANT analogs at different time points (2, 8, 24, 72, 168, 240, and 336 h). Next, we conducted a weighted gene co-expression network analysis (WGCNA) to recognize vital gene modules, and gene set enrichment analysis (GSEA) was further utilized to discover the possible mechanism in the progress of AIC. This study captured the expression alterations of ORMFs during Dox treatment and Epi and Ida treatment. Furthermore, therapeutics for AIC have not been established. Our study might contribute to innovative alternative therapies for AIC.

## Materials and methods

### Data sources

The RNA expression data of the iPSC-derived human cardiac microtissue cell mode and the cardiac biopsies obtained from heart failure patients are publicly accessible at the Hepatic and Cardiac Toxicity Systems modeling project funded by the European Union Seventh Framework Programme (FP7/2007–2013). Cardiac microtissues improve the specificity and sensitivity of assays designed to estimate cardiac safety risks of screening chemicals. The human cardiac microtissues used in the study (3D InSightTM Human Heart Microtissues from InSphero) contained 4,000 iPS-derived human cardiomyocytes from a female Caucasian donor and 1,000 cardiac fibroblasts from a male Caucasian donor. The microtissues were cultured in 3D InSightTM Human Heart Microtissue Maintenance Medium (InSphero) and treated with Dox, Epi, and Ida for two weeks, which were dissolved in 0.1% DMSO. The sample medium was refreshed three times, corresponding to 2, 8 and 24-hour drug concentration distributions.The probes were converted into corresponding gene symbols after the log2 transformation of expression values. For each treatment and dose time-resolved data, MaSigPro [25], running with a polynomial regression model using condition and time postinjection as predictor variables, was used to identify genes/pathways that exhibit differential temporal response between treatment and control conditions.

GSE59672 and GSE2965 were collected to verify the expression levels of the key genes .GSE59672 consists of three wild-type littermates treated with Dox and three control samples. Dox was injected intraperitoneally at a single dose of 20 mg/kg. On day 5, whole hearts were selected for RNA extraction. GSE2965 consists of 8 mice that were randomly assigned to either a control or Dox treatment group. The treated mice received 3 mg/kg Dox weekly for 12 weeks. The hearts of treated and control mice were harvested at day 1 and at 1, 3, 6, 12, and 18 weeks after the first injection.

### Preparation of oxidative stress - mitochondrial dysfunction - ferroptosis-associated gene set

Genes involved in oxidative stress were obtained from the GeneCard database(https://www.genecards.org/) using the “oxidative stress”. Mitochondrial human genes were obtained from the MitoCarta3.0 database (http://www.broadinstitute.org/mitocarta). Ferroptosis-associated genes were obtained from the FerrDb database (http://www.zhounan.org/ferrdb/current/). This database offers an extensive collection of ferroptosis regulatory markers and related diseases.

### Weighted gene co‑expression network analysis (WGCNA)

Using the R WGCNA package, we conducted WGCNA to identify modules of highly correlated genes [26], according to the normalized and transformed gene expression data across samples extracted from the iPSC-derived human cardiac microtissues cell mode. Hierarchical clustering sample trees performing cluster analysis were drawn to detect outliers and remove abnormal samples.

An adjacency matrix was created based on Pearson’s correlations to compare transcript expression levels in the dataset. The pickSoftThreshold in-package function selected the appropriate soft-thresholding power to provide a scale-free topology index (R^2^) of at least 0.9. The adjacency matrix was transformed into the topological overlap matrix (TOM), which represents the degree of shared neighbors between pairs of transcripts. Using the DynamicTreeCut algorithm setting deepSplit = 2, minModuleSize = 30, the gene dendrogram created from TOM and module color were obtained and allocated to colors [26]. Module eigengenes (MEs), which recapitulate the manifestation of genes of a specific module into a single characteristic expression profile, were used to define ANTs-affected modules. Only modules with the strongest relativity were identified for subsequent analysis. Within each module, hub-genes (highly connected genes that likely drive a module’s function) were designated based on the ME-based connectivity measure (KME), which is the distance from the expression profile of a gene to that of the ME. The shared genes of the hub genes and the dynamic response genes were used for subsequent analysis.

### Functional enrichment analysis

The clusterProfiler package was utilized to conduct Gene Ontology (GO) enrichment analysis and Gene set enrichment analysis (GSEA). Gene Set Variation Analysis (GSVA) package was applied in Single sample gene enrichment analysis (ssGSEA) to analyze immune cell infiltration and the angiogenesis score. Adjust P-value < 0.05 was considered statistically significant. Metagene of the 28 immune cells, including activated CD4 T cell, activated B cell, activated CD8 T cell, activated dendritic cell, CD56 bright natural killer cell, CD56dim natural killer cell, central memory CD4 T cell, effector memory CD4 T cell, central memory CD8 T cell, effector memory CD8 T cell, gamma delta T cell, macrophage, eosinophil, immature B cell, immature dendritic cell, mast cell, MDSC, memory B cell, monocyte, neutrophil, plasmacytoid dendritic cell, regulatory T cell, T follicular helper cell, natural killer cell, natural killer T cell, Type 1 T helper cell, Type 2 T helper cell, and Type 17 T helper cell, were obtained from a published study [27]. The angiogenesis-relevant data were acquired from the MSigDB Team (Hallmark Gene set).

### Molecular docking of anthracyclines with key genes

The 3-dimensional conformer of Anthracyclines were downloaded from PubChem databases (https://pubchem.ncbi.nlm.nih.gov/) and proteins that are related to the key genes were downloaded from the RCSB Protein Data Bank. The preparation of the Dox and proteins and the molecular docking was carried out using Autodock Vina 1.5.6 software (http://autodock.scripps.edu/).

## Results

### Sample information

**Fig. 1:**
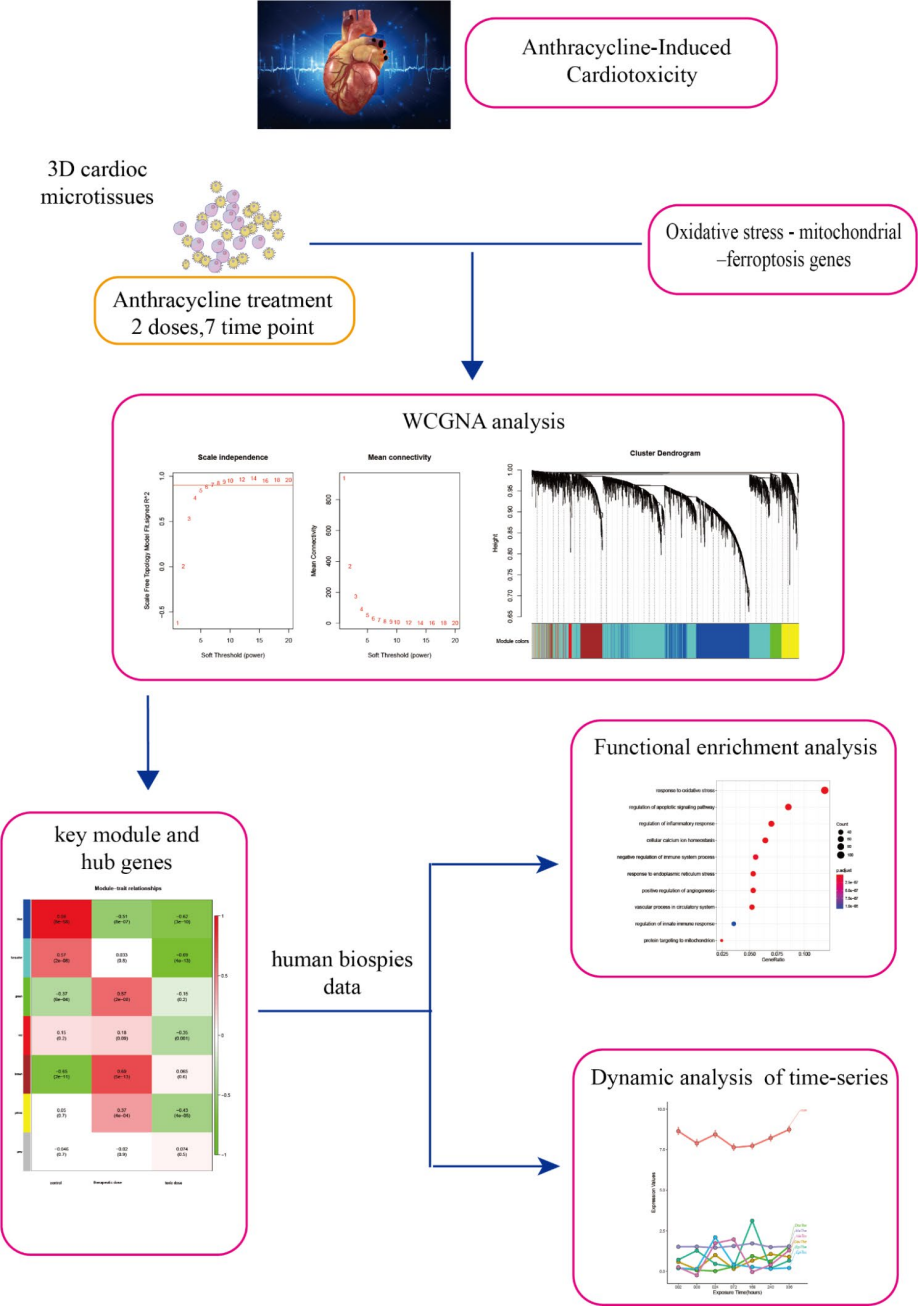
Flow chart for the comprehensive analysis of oxidative stress - mitochondrial dysfunction - ferroptosis genes and pathways in anthracycline-induced cardiotoxicity

A workflow of the analyses is shown in Fig. 1.

The iPSC-derived human cardiac microtissue cell mode was designed to resemble the 2-based repetitive dosing profile, in particular, a therapeutic dose and a toxic dose, for 2 weeks. The therapeutic dose simulates processes analogous to the phenomenology of delayed chronic cardiotoxicity, while the toxic dose simulates acute cardiotoxicity [28]. The microtissues were exposed to three anthracycline drugs (Dox, Epi, and Ida), and the fluctuation in interstitial cardiac tissue concentration throughout time was rectified using reversed pharmacokinetic modeling [29]. After exposure to ANTs for 2, 8, 24, 72, 168, 240, and 336 h, the transcriptome responses were assessed, with each time point measured in triplicate. Due to significant cell death, the Ida toxic-treated samples at 240 and 336 h were disregarded. The study acquired cardiac biopsies from 18 heart failure patients, including 7 controls with no cancer history, 10 patients with cancer who received ANTs, and 1 with cancer who received medication without ANTs.

### WGCNA and identification of the key module

First, 844 ferroptosis-associated genes, 1136 mitochondrial function-associated genes, and 4274 oxidative stress-associated genes were defined. Three sets of genes were then intersected with the in vitro dataset and biopsies dataset, identifying a total of 3412 overlapping ORMFs in both data sets, including 425 ferroptosis-associated genes, 1046 mitochondrial function-associated genes, and 2708 oxidative stress-associated genes (Figure S1).

**Fig. 2:**
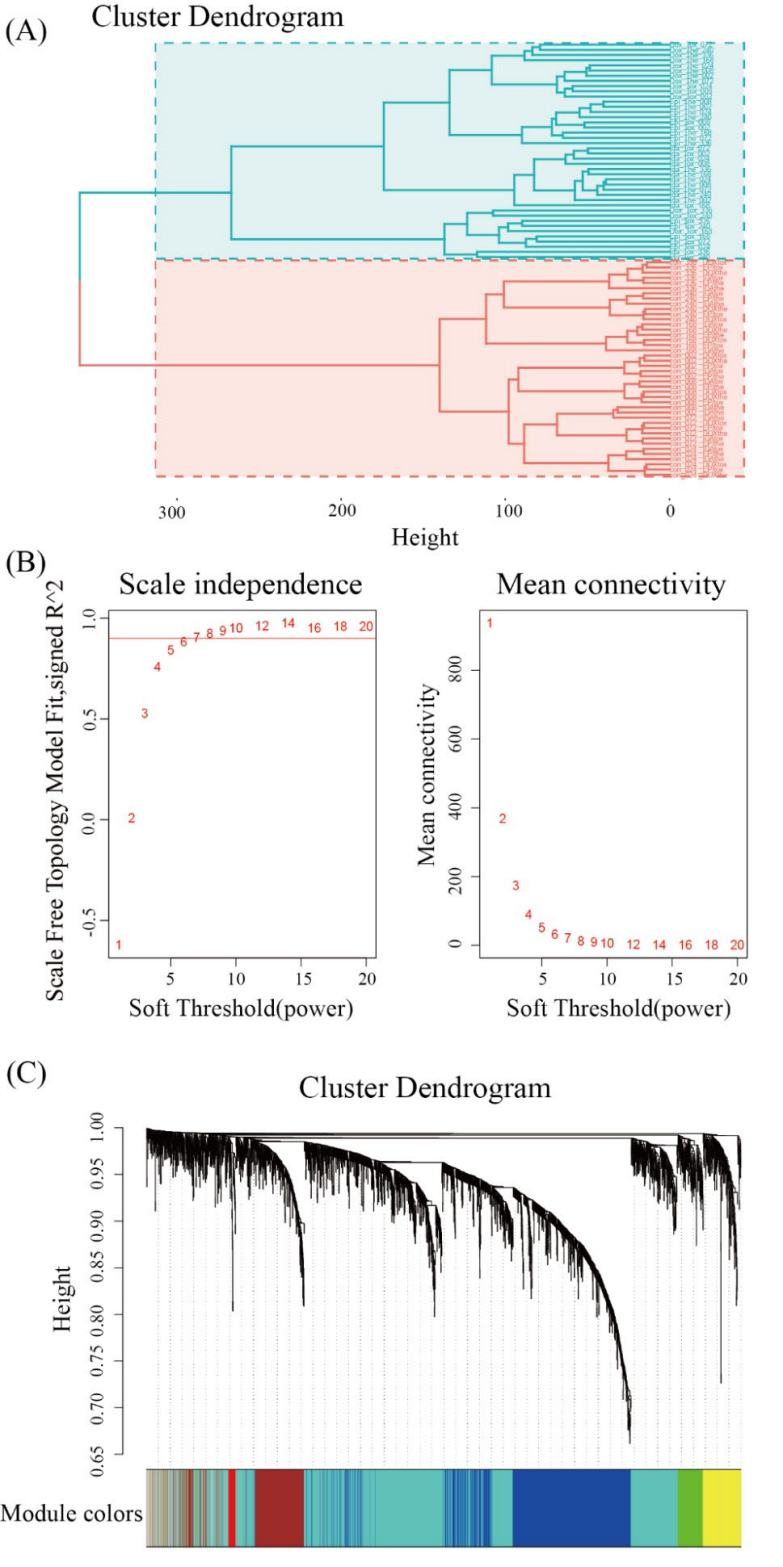
Clustering of samples and detection of soft-thresholding power. (**A**) The hierarchical clustering tree was conducted to filter outliers. All samples are distributed in clusters, with controls scattered among the red branches of the cluster tree. (**B**) Analysis of the scale-free fit index and the mean connectivity for numerous soft-thresholding powers. (**C**) Dendrogram of all genes organized corresponding to a dissimilarity measure (1-TOM), where each branch represents a gene, and low-hanging clusters represent gatherings of genes (modules) with roughly comparable network attributes (branches) on the tree

In vitro ORMF transcriptome profile clustering tree with samples split into two major groups and all control samples equally distributed along one branch (Fig. 2A).

**Fig. 3:**
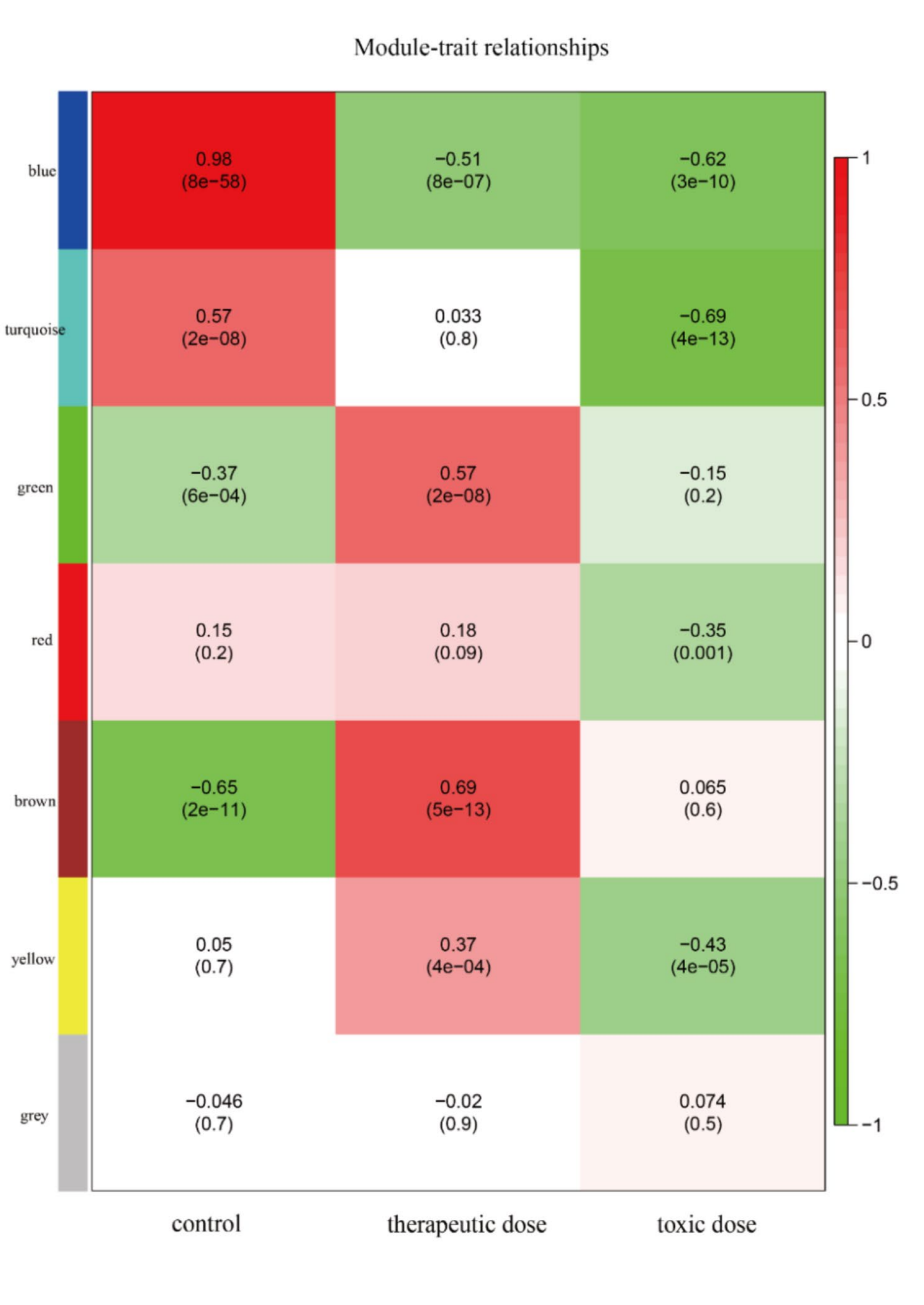
Heatmap of module-trait relationships displaying associations between module eigengenes and clinicopathological characteristics. A module eigengene is represented by each row, while clinicopathological characteristics are represented by each column. Numbers in the table refer to the correlation r and the P value in parentheses. The color legend illustrates the degree of correlation. Red and green colors indicate positive or negative relationships

In order to uncover the responsive modules and hub genes in the adverse mechanisms of anthracycline cardiotoxicity, a weighted gene co-expression network was constructed using ORMFs. To construct the scale-free network, we selected β = 6 as the soft threshold (Fig. 2B). A total of 7 modules (Fig. 2C) were obtained based on the scale-free network. Among the modules, the blue module was strongly associated with in vivo drug exposure levels (Fig. 3). As a result, the blue module served as the hub module. The blue module contained 931 genes.

### Selection of key genes and biological processes

As demonstrated by their high KME (eigengene connectivity) value, hub genes have the most connections in the network. Therefore, 17 genes with KME ≥ 0.95, including 1 ferroptosis-associated gene, 2 mitochondrial function-associated genes, and 16 oxidative stress-associated genes, were identified as hub genes.

After investigating the intersection of hub genes and the dynamic response genes, 8 genes related to AIC progression were obtained: CD36 Molecule (CD36), Cadherin 5 (CDH5), Chitinase 3 Like 1 (CHI3L1), Hemoglobin Subunit Alpha 2 (HBA2), Hydroxysteroid 11-Beta Dehydrogenase 1 (HSD11B1), Osteoglycin (OGN), Ribosomal Protein L8 (RPL8), and Von Willebrand Factor (VWF).

**Fig. 4:**
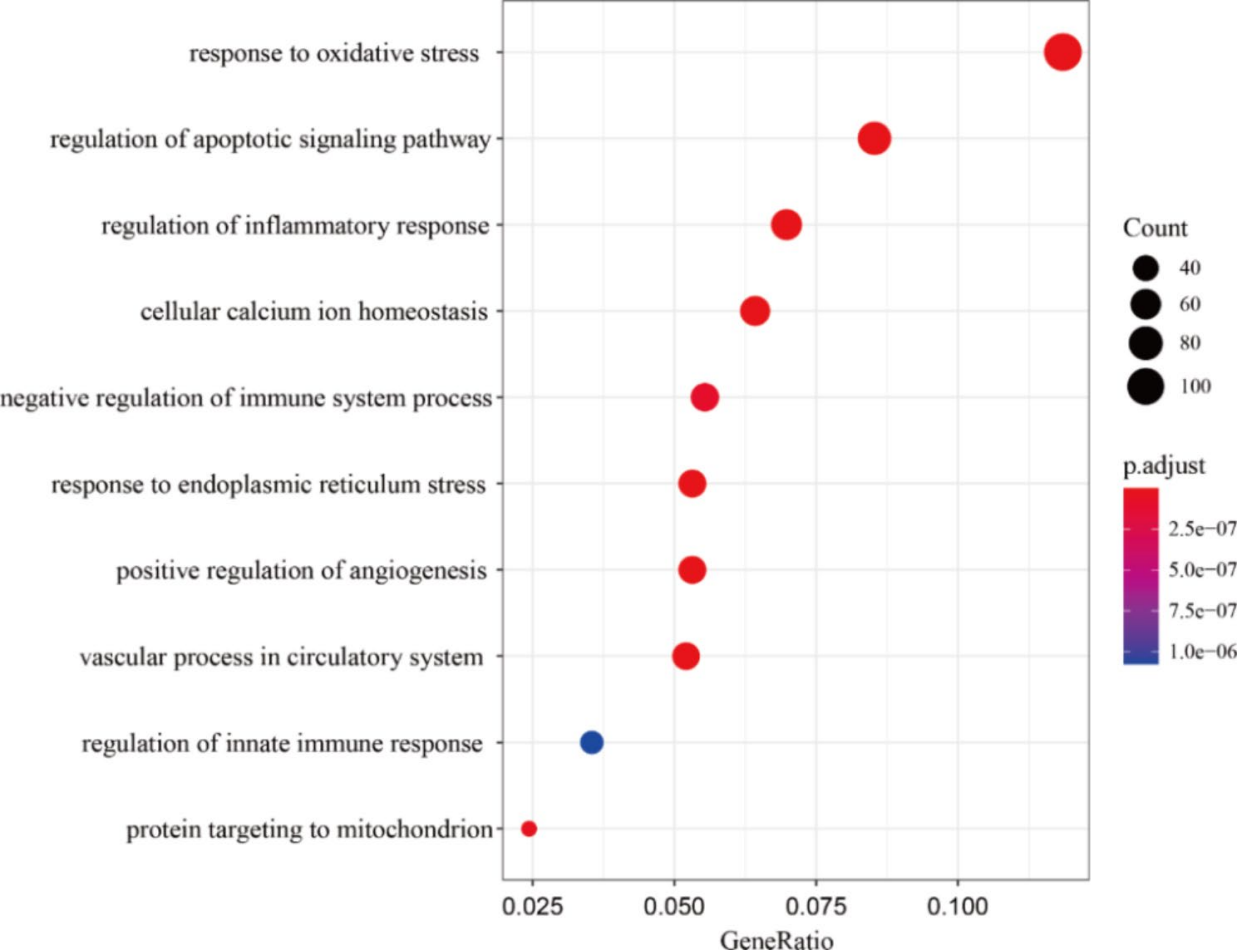
The results of GO enrichment analysis based on biological processes (BP) of blue module genes

In order to identify biological functions and the pathways associated with the adverse mechanisms of AIC, we further conducted enrichment analysis for genes in the blue module. Gene Ontology (GO) enrichment analysis indicated that enriched biological functions and pathways related to oxidative stress and mitochondrial function were significantly enriched. Surprisingly, the results revealed that pathways pertaining to angiogenesis and the immune system, namely GO:0045766 positive regulation of angiogenesis and GO:0002683 negative regulation of immune system process, were both engaged and then chosen for more in-depth research (Fig. 4, Table S1).

### Expression of the key genes and the biological processes

**Fig. 5:**
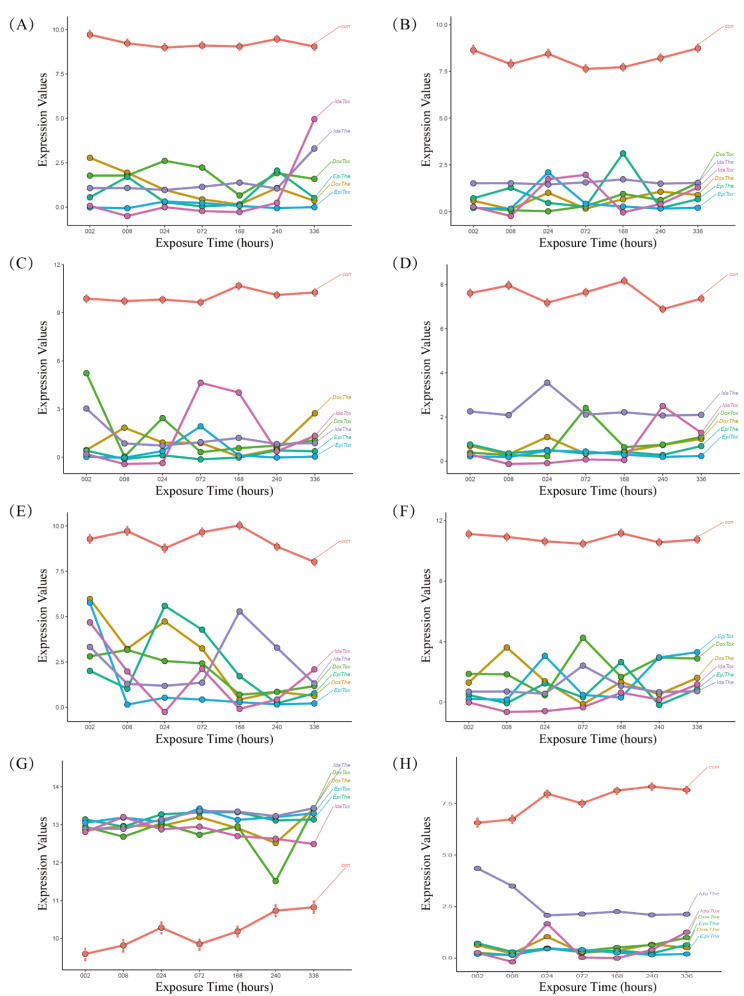
The dynamic alterations of the key genes characterized log2FC expression values at each time between anthracycline (ANT) and control samples. (**A**) CD36, (**B**) CDH5, (**C**) CHI3L1, (**D**) HBA2, (**E**) HSD11B1, (**F**) VWF, (**G**) RPL8, (**H**) OGN. The: therapeutic dose, Tox: toxic dose; 002, 008, 024, 072, 168, 240, 336 are corresponding exposure periods in hours; con: the control group; Dox: doxorubicin, Ida: idarubicin; Epi: epirubicin

**Fig. 6:**
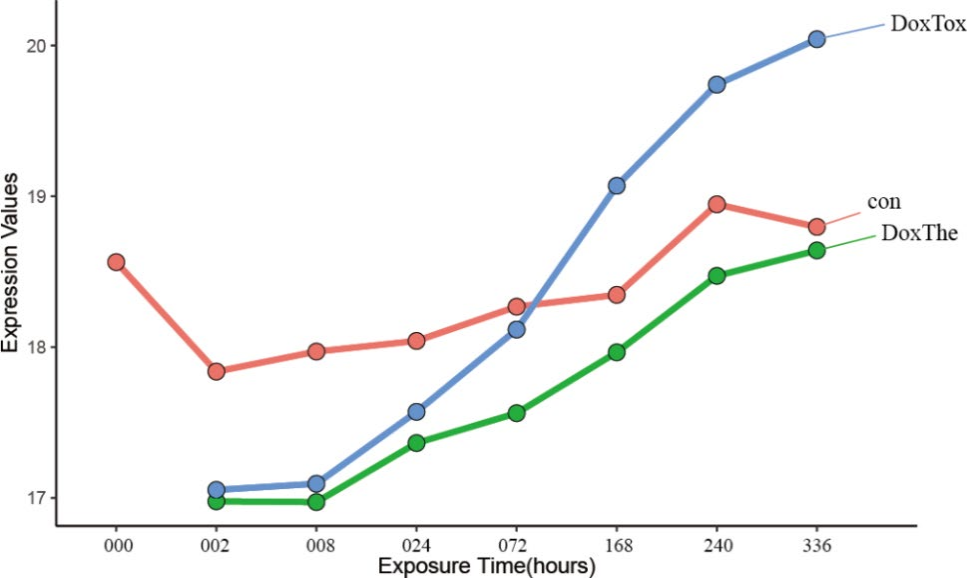
The dynamic alterations of Hemoglobin subunit alpha (HBA) characterized log2FC expression values at each time between doxorubicin (Dox) and control samples. The: therapeutic dose, Tox: toxic dose; 002, 008, 024, 072, 168, 240, 336 are corresponding exposure periods in hours; con: the control group; Dox: doxorubicin

All 8 key genes were consistently differentially expressed across all ANT-treated conditions, and their expression levels varied over time. The expression of all the important genes, except RPL8, was reduced in the conditions treated with ANT in vitro. (Fig. 5). Notably, the protein level of Hemoglobin subunit alpha (HBA) encoded by HBA2 varied over time under conditions of Dox treatment in vitro (Fig. 6).

**Fig. 7:**
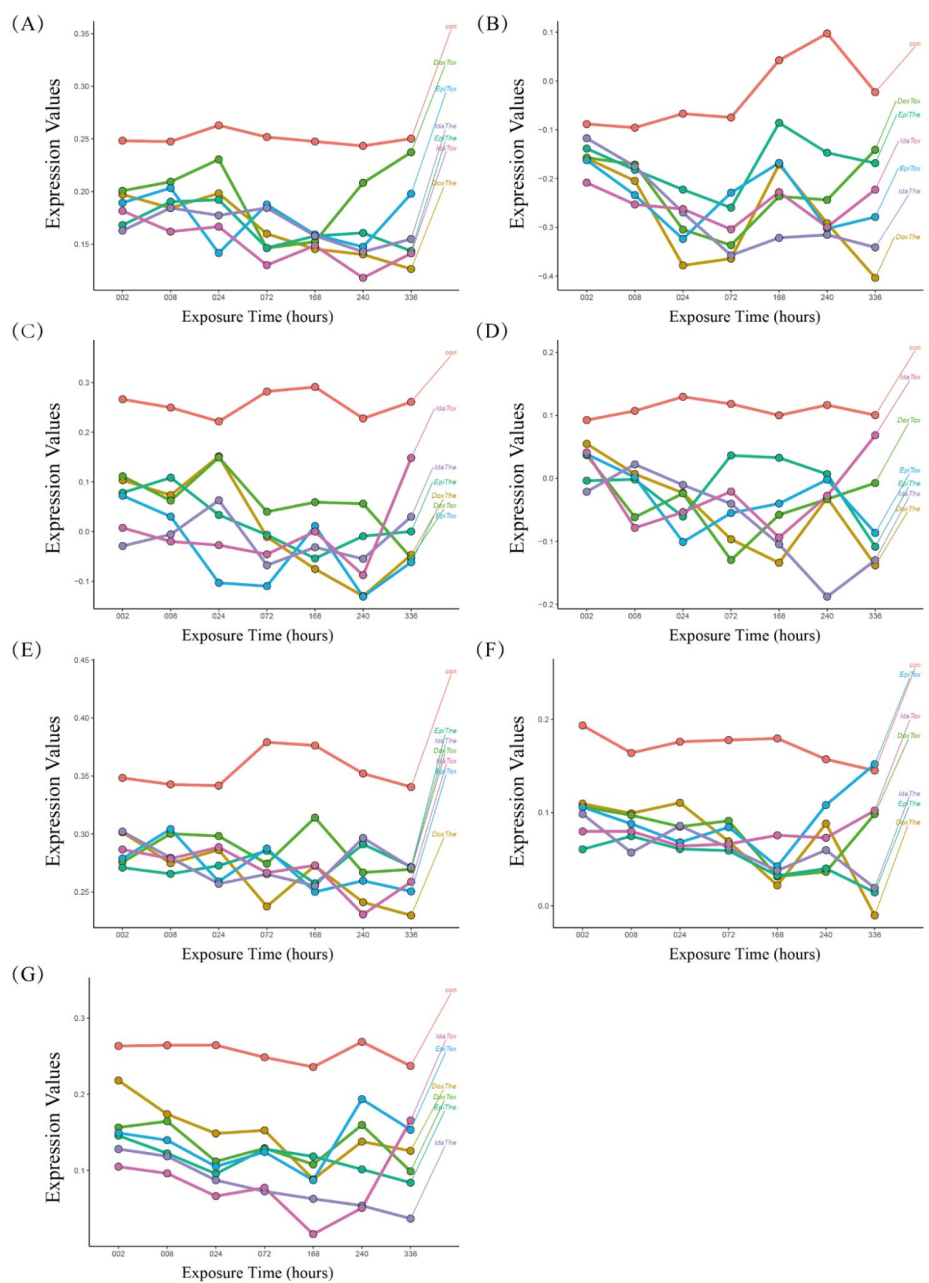
The dynamic alterations of the angiogenesis score and the immune cells were characterized by comparing the expression levels of anthracycline (ANT) samples with control samples at each time point. (**A**) The angiogenesis score, (**B**) Activated CD4 T cell, (**C**) Immature B cell, (**D**) Memory B cell, (**E**) Natural killer cell, (**F**) Type 1 T helper cell, (**G**) Type 2 T helper cell. The: therapeutic dose, Tox: toxic dose; 002, 008, 024, 072, 168, 240, 336 are corresponding exposure periods in hours; con: the control group; Dox: doxorubicin, Ida: idarubicin; Epi: epirubicin

The quantitatively assessed angiogenesis score for each sample in the current study was calculated. Single-sample gene set enrichment analysis (ssGSEA) was also employed to assess the degree of immune cell infiltration in each sample. The dynamic characteristics of the angiogenesis score and the immune cells were also analyzed in the present study to identify dynamically altered pathways. The angiogenesis score and immune cell types, like activated CD4 T cells, immature B cells, memory B cells, natural killer cells, type 1 T helper cells, and Type 2 T helper cells, were down-regulated and showed a remarkable alteration across all ANT-treated conditions (Fig. 7**)**.

**Fig. 8:**
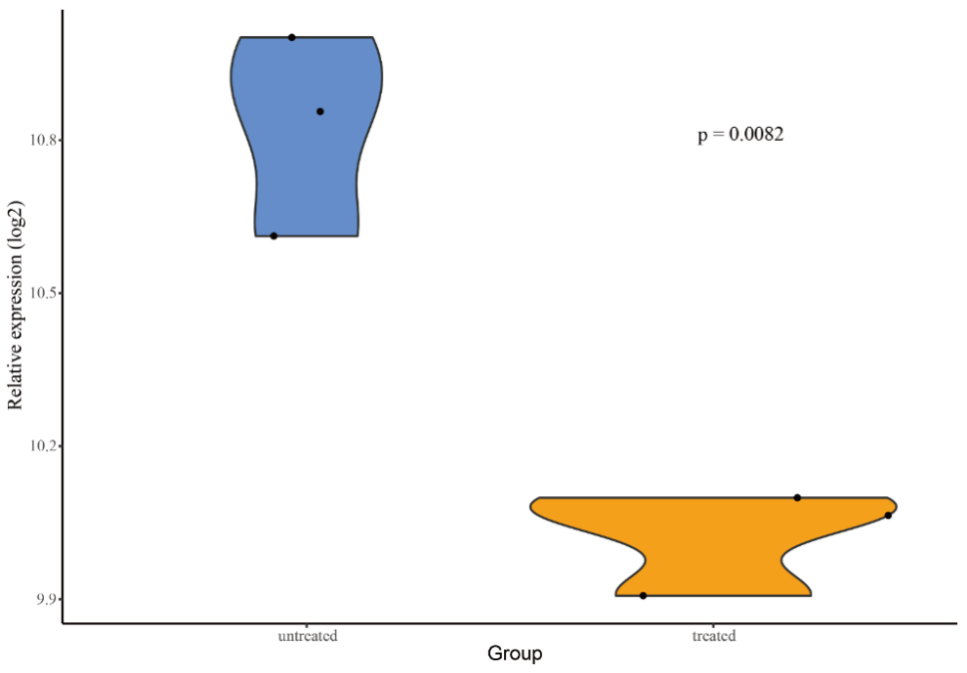
The expression of OGN was dysregulated in GSE59672

**Fig. 9:**
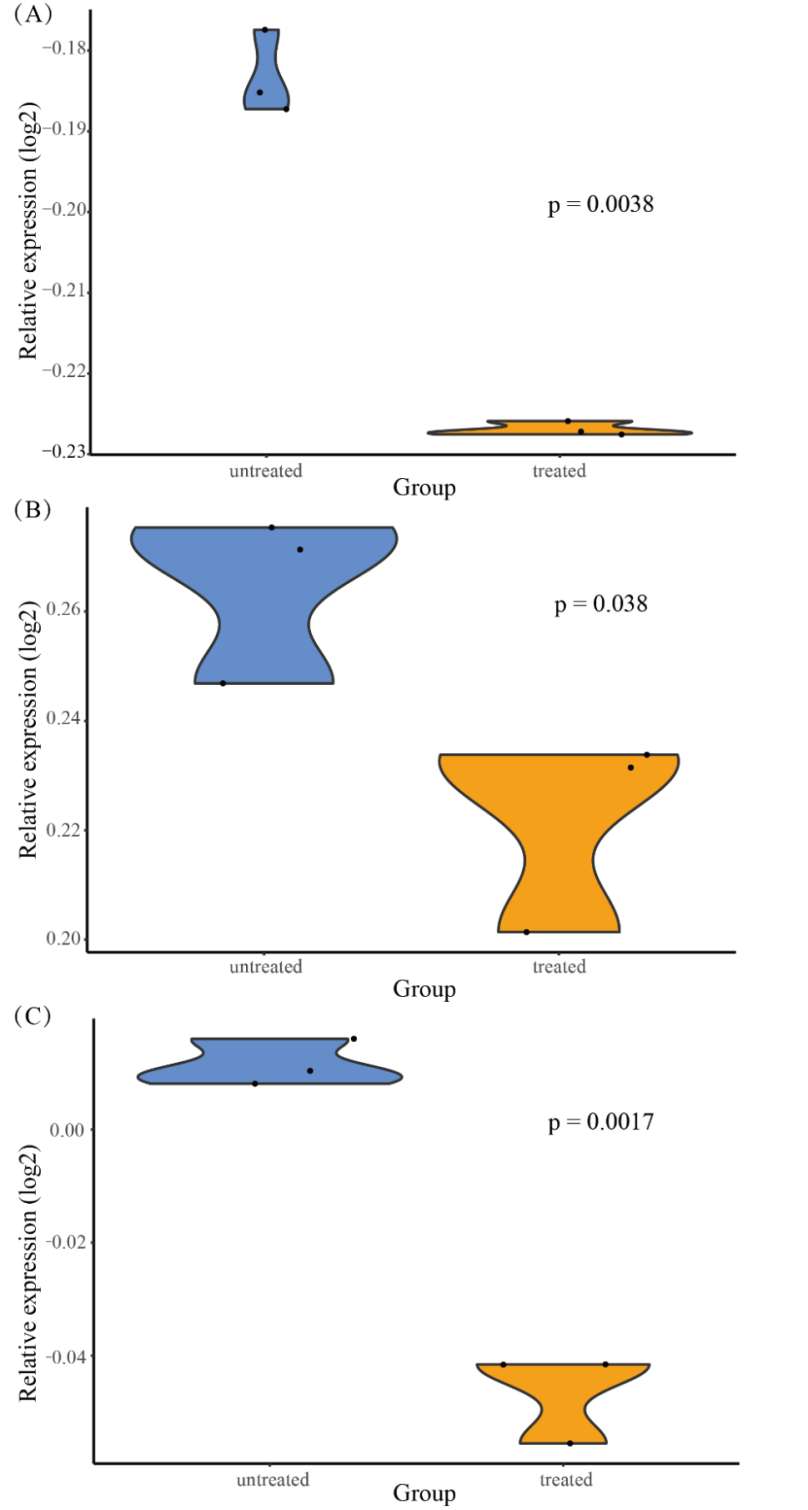
The immune cell types of immature B cells, natural killer cells and Type 2 T helper cells were dysregulated in GSE59672. (**A**) immature B cells (**B**) natural killer cells (**C**) Type 2 T helper cells

**Fig. 10:**
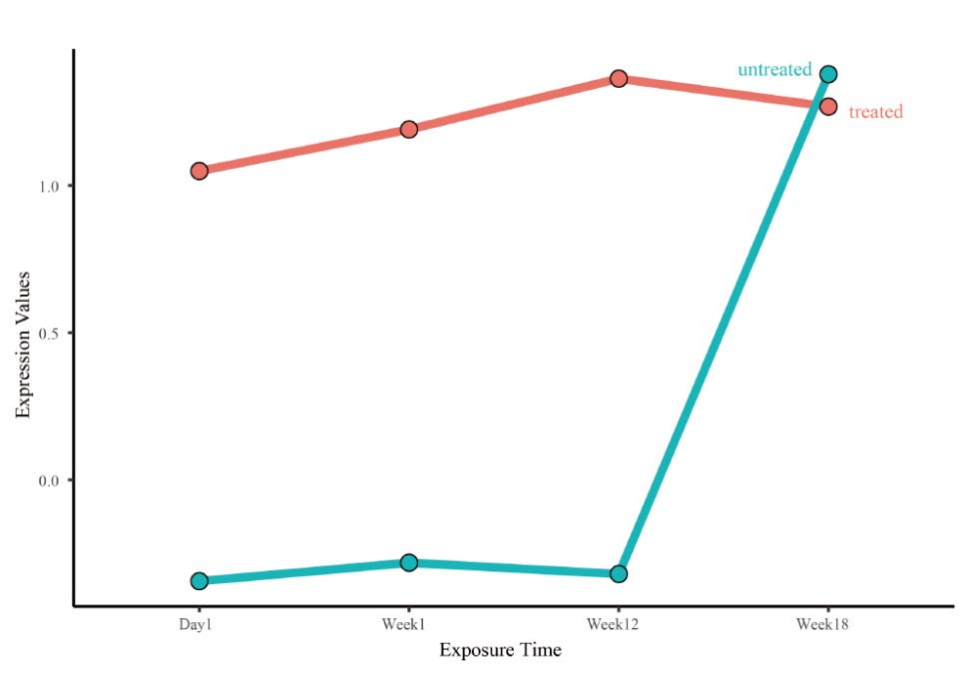
The expression level of RPL8 varied over time in GSE2965

**Fig. 11:**
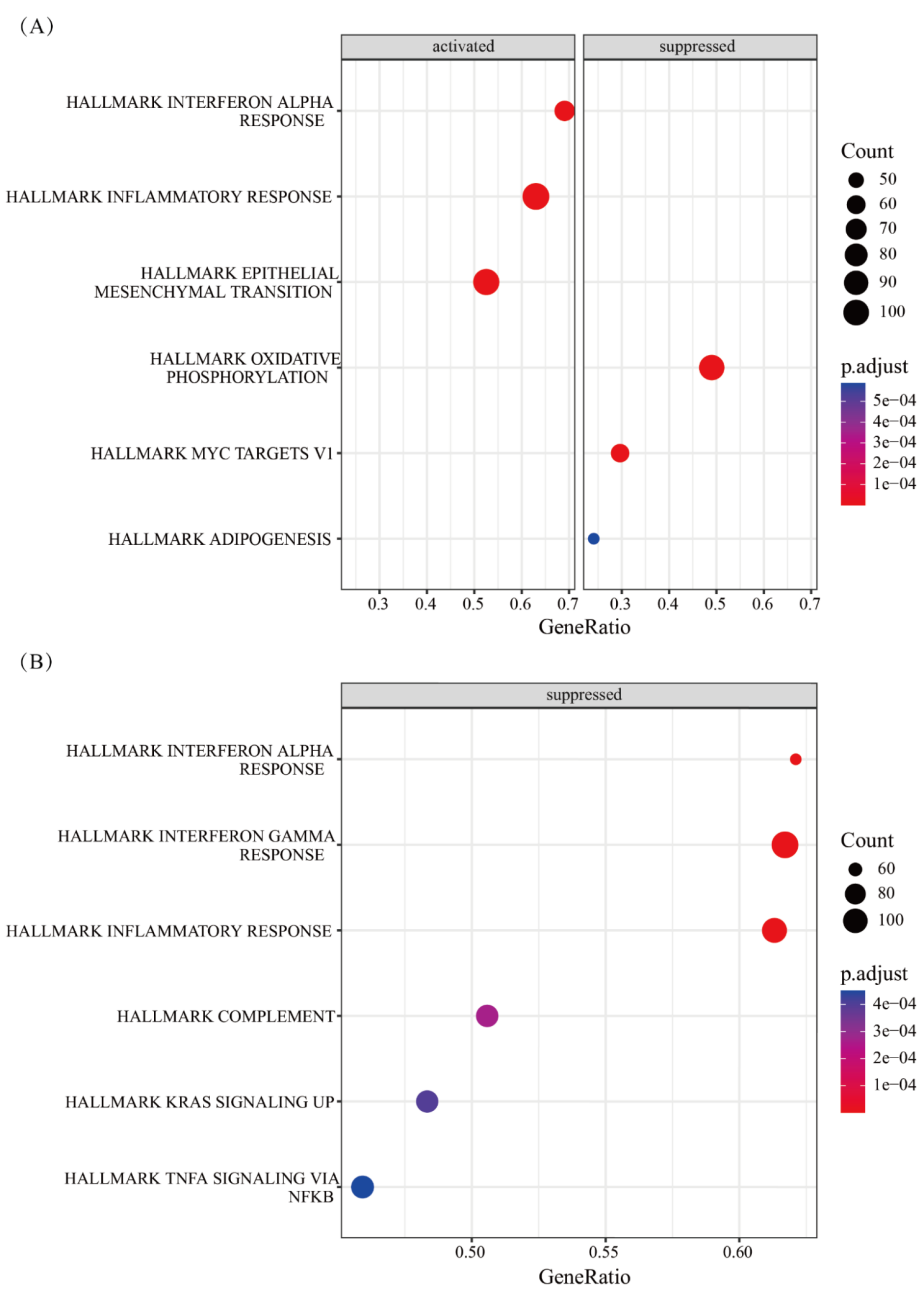
The pathways correlated with CHI3L1 and RPL8. (**A**) CHI3L1 (**B**) RPL8

In the human heart biopsy samples, there were no statistically significant differences in the expressions of key genes, immune cells, and angiogenesis score between cancer patients who received ANT and non-ANT treatment vs. non-cancer patients. In GSE59672, the expression of OGN (Fig. 8) and the immune cell types of immature B cells, natural killer cells and Type 2 T helper cells (Fig. 9) were dysregulated. In GSE2965, the expression level of RPL8 varied over time (Fig. 10). Notably, due to the limited number of probes, GSE2965 has only the key genes HSD11B1, CD36, RPL8 and HBA2, while pathway expression could not be analyzed. To explore the potential mechanism of RPL8 and CHI3L1 in AIC, Fig. 11 shows the pathways predicted by GSEA.

### Molecular docking of anthracyclines with the key genes

**Fig. 12:**
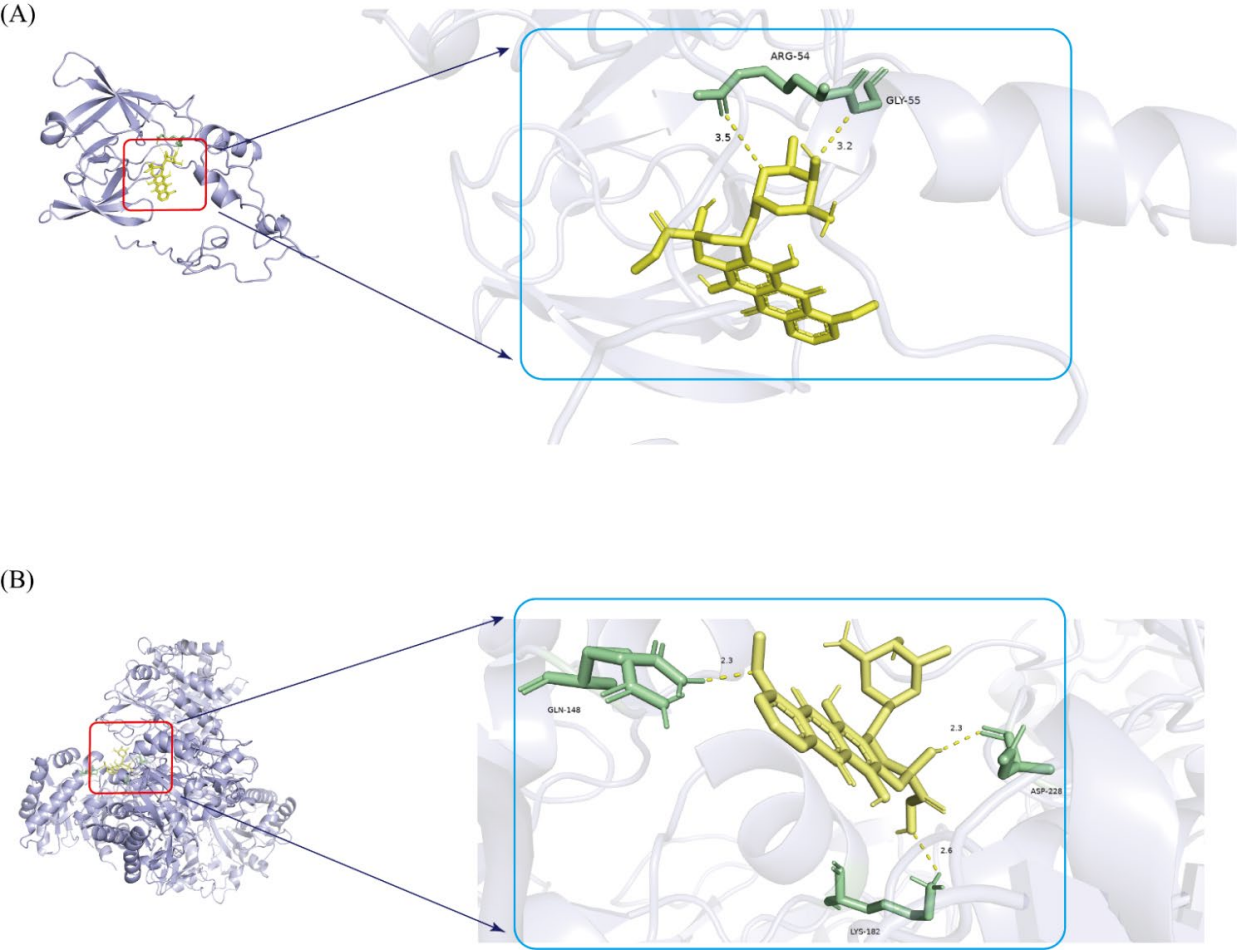
The 3D diagrams of Dox and its binding to RPL8 and CHI3L1. (**A**) RPL8 (**B**) CHI3L1

Molecular docking was used to to characterize the interaction of anthracyclines with key genes. The protein product of RPL8 and CHI3L1 genes showed significant binding affinity for anthracyclines (binding affinity < -4.8). Notably, GSE2965 did not detect CHI3L1 expression due to the limited number of probes, whereas GSE59672 had a small number of samples, and the expression of RPL8 and CHI3L1 was different but not statistically significant (Figure S2). The amino acid residues of RPL8 and CHI3L1 interacting with doxorubicin are shown in Fig. 12. Gene names, protein database IDs and binding affinities are shown in Table S2.

## Discussion

AIC is a multifactorial disorder involving oxidative stress, mitochondrial malfunction, and ferroptosis. A single regulator cannot adequately figure out these factors. ROS generation and release are frequently associated with ferroptosis, which may result in mitochondrial dysfunction. The effect of ANTs on ORMFs was investigated in an in vitro cardiac microtissue experiment and heart failure biopsies from patients treated with ANTs in this study. The dynamic alterations of these genes and their cellular mechanisms were appraised. Our study is the first to employ ORMFs to describe the time course of ANTs-induced cardiotoxicity and guide effective therapy.

These key genes could be biological indicators for cardiotoxicity and provide a novel understanding of the dynamic alterations caused by ANTs. One of the key genes was VWF, an acceptable biomarker of endothelial dysfunction in vascular diseases [30]. VWF, elevated throughout chemotherapy, particularly in individuals with vascular disorders [31], could be used as a diagnostic strategy for early diagnosing vascular diseases in chemotherapy-treated cancer patients [32]. CD36 (fatty acid translocase), coordinating myocardial lipid metabolism by modulating lipid signaling and promoting the metabolism of long-chain fatty acids on cardiomyocytes, is upregulated in models for advanced cancers. In the therapy of AIC, CD36 is a significant target. Our results are inconsistent with the reported [31, 32], with decreased VWF and CD36 expression relative to controls. VWF were assessed before and until 1 year after chemotherapy in metastatic testicular cancer patients cured with bleomycin, etoposide and cisplatin. VWF increased during chemotherapy, especially in patients with vascular events. We hypothesize that the reason for the inconsistency are as follows: Cardiotoxicity due to ANTs is dose-dependent and might manifest immediately or take time to manifest. Variable doses of ANTs and study endpoints across studies, which interfere with the quantitative assessment of genes. Additionally, many animal and human studies focus on whole blood RNA specimens, whereas our study is based on cardiac RAN, and cardiac-based RNA is particularly scarce in human studies.

The function of ORMFs implies the pathways of ANTs, especially the immunological infiltration and the angiogenesis signaling pathway, to be critical issues in the ongoing studies of ANTs-induced cardiotoxicity. Immunity contributed significantly to the occurrence and progression of AIC [33]. Proteins in the blood represent drug-induced immune-mediated reactions and may potentially be sensitive biomarkers of drug-induced cardiotoxicity. High baseline IgE levels at cardiotoxic chemotherapy exposure are related to a lower risk of AIC in breast cancer patients receiving doxorubicin and trastuzumab therapies [34]. One of the hypothesized protective effects of high IgE is that it enhances the induction of T cell-mediated cytotoxicity by stimulating myeloid antigen-presenting cells [35]. Another study identified that breast cancer patients showed elevated plasma levels of cytokines CCL27 and macrophage migration inhibitory factor (MIF) following two cycles of Dox [36, 37]. Dox has been demonstrated to disrupt the immune system through innate immune cells, leading to progressive cardiac damage. Neutrophil infiltration in the heart plays a significant role in cardiac toxicity caused by doxorubicin. In breast cancer patients with Dox-induced cardiotoxicity, a high plasma neutrophil extracellular trap level was seen [38]. Another study confirmed that neutrophil elevation in cardiac tissue reached its highest point seven days following a single bolus of Dox administration [39]. Mechanisms of neutrophil-induced acute interstitial cystitis (AIC) include disrupting vascular structures like pericytes and endothelial cells and increasing collagen deposition [40]. Macrophage undergoing marked phenotypic and functional changes has been associated with AIC evolution. Dox-induced cardiotoxicity affects the balance of circulating M1 pro-inflammatory macrophages recruited from monocytes and M2 anti-inflammatory macrophages engaged in tissue repair [41]. The M1 macrophage population regulating the development of cardiac injury was elevated [42, 43], while the M2 macrophage population was suppressed in Dox-induced acute cardiotoxicity mice models [42, 44, 45]. Immunomodulatory strategies to reverse cardiac M1/M2 macrophage imbalance due to Dox-induced transfer of mitochondria, gut microbiota modulation, inflammation, oxidative stress, apoptosis, and autophagy, may prevent the doxorubicin-induced cardiotoxicity [46–49]. Curiously, our study did not screen for the above immune cells, only showing dynamic changes of Activated CD4 T cell, Immature B cell, Memory B cell, Natural killer cell, Type 1 T helper cell, and Type 2 T helper cell over time and there are few reports about these cells. These novel findings suggest the immune system is a prospective regulator of AIC. However, the role of the immune system in AIC requires a more detailed study.

Regarding the toxic mechanisms of AIC, vascular homeostasis, and angiogenesis have received little attention [5, 50, 51]. Numerous cells, such as endothelial cells (EC), vascular smooth muscle cells, and fibroblasts, as well as a variety of vasoactive chemicals, such as nitric oxide (NO) and vascular endothelial growth factor (VEGF), and their downstream intracellular signaling pathways, are involved vascular homeostasis and angiogenesis. Vascular endothelial growth factor-B (VEGF-B) regulates the proliferation, sprouting, and migration of ECs and can potentially induce coronary vessel growth and cardiac hypertrophy [52]. Dox therapy, both in vivo and in vitro, demonstrated a considerable effect on reducing the expression of VEGF-B [53]. VEGF-B gene therapy prevented Dox-induced DNA damage and mitochondrial dysfunction, increased left ventricular volume, and did not stimulate tumor growth In Dox-treated mice [51]. VEGF-B may be a suitable and powerful treatment for AIC, in line with previous studies demonstrating its benefits in various heart disease models [54–56]. Dox-induced cardiotoxicity was further demonstrated to trigger endothelial damage by reducing the release and activity of critical endothelial factors leading to endothelial cell death, disrupting the endothelial barrier function, and increasing vascular permeability. This can precipitate the onset and progression of cardiomyopathy [57, 58]. Therefore, the biochemical and molecular properties of vascular homeostasis undergoing AIC therapy must be considered when developing therapeutic drugs to comprehensively treat the entire cardiovascular system.

This study has several limitations that need to be addressed. Firstly, there was no statistical difference in key gene expression, immune cells, and angiogenesis scores between cancer patients and non-cancer patients receiving ANT and non-ANT treatment, which could be attributed to the limited quantity of human samples, and the specific reasons need to be further elucidated by external data sets. Secondly, quantitative studies at the microtissue cellular level, such as key gene expression and immune cell infiltration, are inconsistent with the reported results of animal and human specimens, and further research is needed. Thirdly, although our study shows that AIC is related to angiogenesis, further research is required to elucidate the specific cells and pathways involved in this mechanism.

## Conclusion

Increasing evidence shows that ORMFs have an indispensable role in AIC. Our study found that the dynamic alternation of oxidative stress - mitochondrial dysfunction - ferroptosis genes may impact cardiac immune functions and angiogenesis. These findings implicate an angle that warrants thorough investigation.

### Electronic supplementary material

Below is the link to the electronic supplementary material.

Supplementary Material 1

Supplementary Material 2

Supplementary Material 3

## Data Availability

The data presented in the study are deposited at the Hepatic and Cardiac Toxicity Systems modeling project funded by the European Union Seventh Framework Programme (FP7/2007–2013). Further inquiries can be directed to the corresponding author.

## Abbreviations

ORMFs: Oxidative stress - mitochondrial dysfunction – ferroptosis
WGCNA: Weighted gene correlation network analysis
ssGSEA: Single sample Gene Set Enrichment Analysis
ANTs: Anthracyclines
AIC: Anthracycline-induced cardiotoxicity
Dox: Doxorubicin
Epi: Epirubicin
Ida: Idarubicin
ROS: Reactive oxygen species
Mn-SOD: Manganese superoxide dismutase
GPx1: Glutathione peroxidase 1
BH4: Tetrahydrobiopterin
GSH: Glutathione
PLOOH: Phospholipid hydroperoxides
NRF2: Erythroid 2-related factor 2
TOM: Topological overlap matrix
MEs: Module eigengenes
GO: Gene Ontology
GSVA: Gene Set Variation Analysis
KME: Hub-genes were designated based on the ME-based connectivity measure
HBA: Hemoglobin subunit alpha
MIF: Macrophage migration inhibitory factor
NO: Itric oxide
EC: Endothelial cells
VEGF: Ascular endothelial growth factor
