# Supplementary material for: A longitudinal evaluation of oxidative stress - mitochondrial dysfunction - ferroptosis genes in anthracycline-induced cardiotoxicity
Source: BMC Cardiovasc Disord. 2024 Jul 10;24:350. doi: 10.1186/s12872-024-03967-z (PMC11234563; doi:10.1186/s12872-024-03967-z)

**SUPPLEMENTARY FILE**

**Title: A Longitudinal Evaluation of Oxidative Stress - Mitochondrial Dysfunction - Ferroptosis Genes in Anthracycline-Induced Cardiotoxicity**

**Figure S1.** The intersection of ferroptosis-associated genes (Ferroptosis), mitochondrial function-associated genes (Mitochondria), and oxidative stress-associated genes (ROS).

**Figure S2.** The expression of CHI3L1 and RPL8 in GSE59672 (A) CHI3L1 (B) RPL8.

**Figure S1.** The intersection of ferroptosis-associated genes (Ferroptosis), mitochondrial function-associated genes (Mitochondria), and oxidative stress-associated genes (ROS).


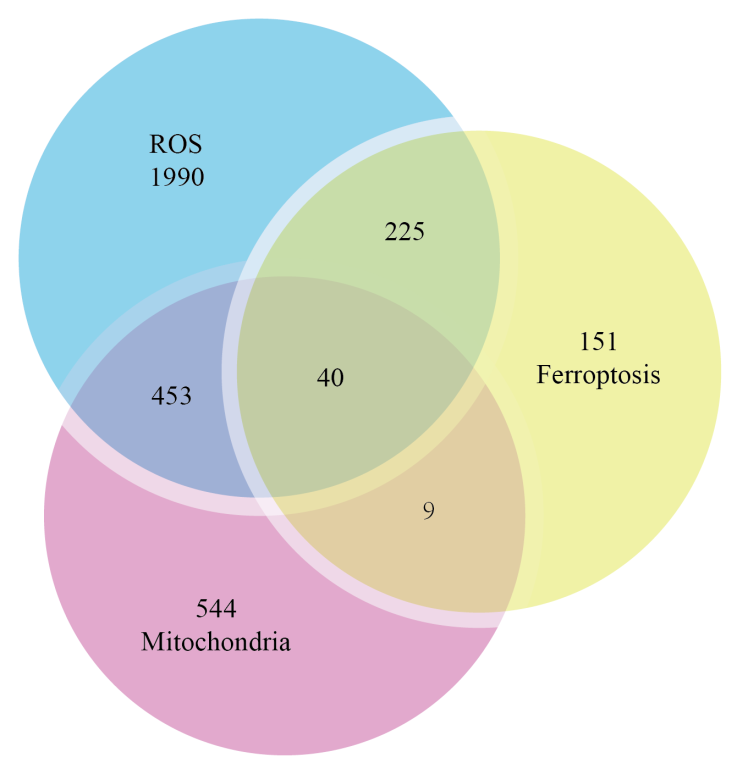


**Figure S2.** The expression of CHI3L1 and RPL8 in GSE59672. (A) CHI3L1 (B) RPL8.


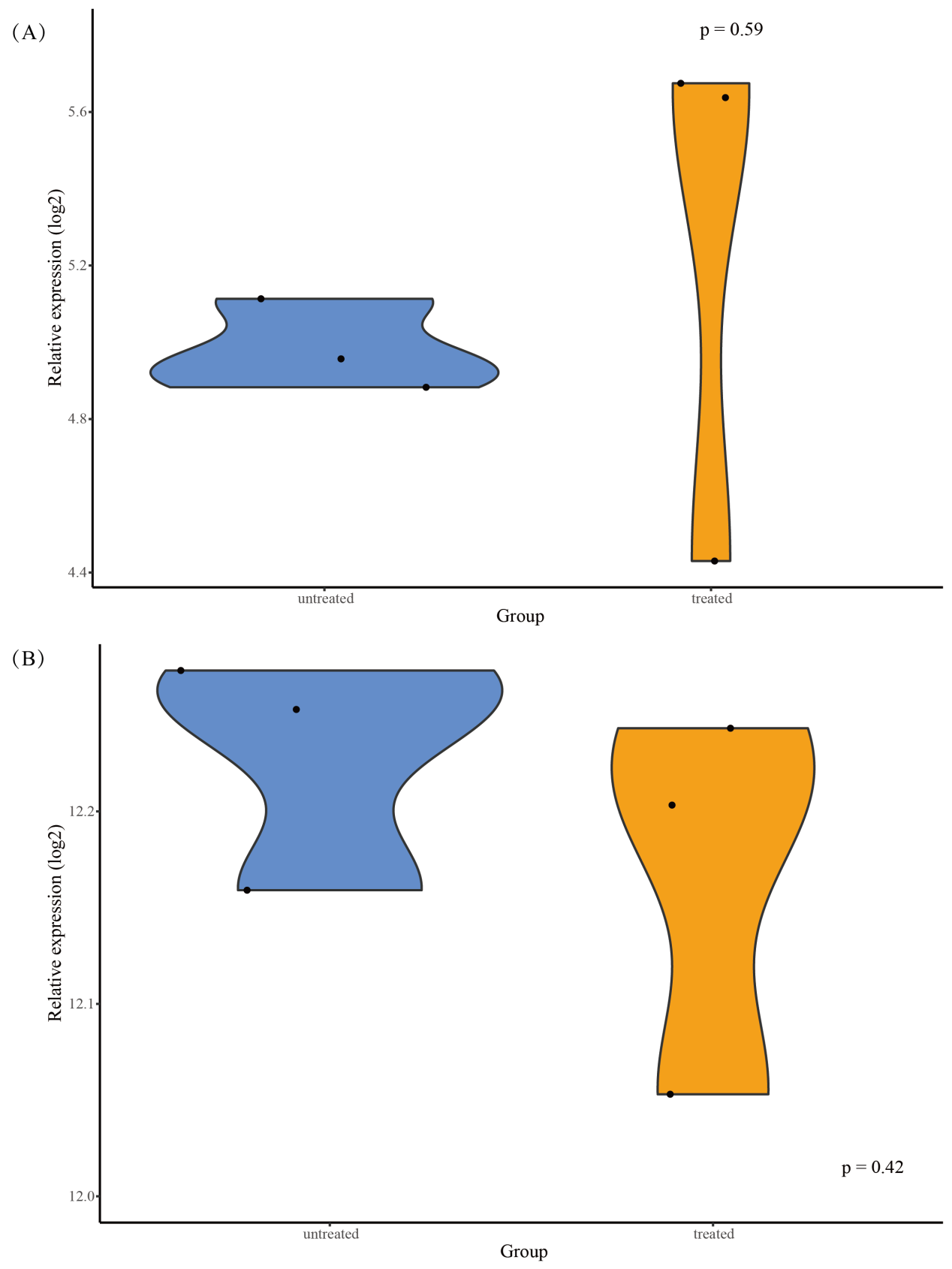

Supplement: Supplementary file 1 — Supplementary Material 1 [file 12872_2024_3967_MOESM1_ESM.docx]
